# Very Late Effects of Postoperative Atrial Fibrillation on Outcome of Coronary Artery Bypass Graft Surgery

**DOI:** 10.5812/cardiovascmed.4584

**Published:** 2012-11-01

**Authors:** Majid Haghjoo, Mona Heidarali, Salman Nikfarjam, Mohammadmahdi Peighambari, Alireza Alizadeh-Ghavidel, Saeid Hosseini, Alireza Jalali

**Affiliations:** 1 Cardiac Electrophysiology Research Center, Rajaie Cardiovascular Medical and Research Center, Tehran University of Medical Sciences, Tehran, IR Iran; 2 Cardiovascular Intervention Research Center, Rajaie Cardiovascular Medical and Research Center, Tehran University of Medical Sciences, Tehran, IR Iran; 3 Heart Valve Disease Research Center, Rajaie Cardiovascular Medical and Research Center, Tehran University of Medical Sciences, Tehran, IR Iran; 4 Department of Cardiac Anesthesiology, Rajaie Cardiovascular Medical and Research Center, Tehran University of Medical Sciences, Tehran, IR Iran

**Keywords:** Atrial Fibrillation, Coronary Disease, Mortality, Morbidity

## Abstract

**Background:**

Atrial fibrillation (AF) after coronary artery bypass graft (CABG) is a common complication with potentially higher risk of adverse outcome and prolonged hospital stay.

**Objective:**

To determine the impact of postoperative AF (POAF) on long-term outcome in a large cohort of patients who underwent CABG.

**Patients and Methods:**

We conducted an observational cohort study of 989 patients who underwent isolated CABG with more than 5-year follow-up. Patient divided in two groups: patients with and without POAF.

**Results:**

In this study, atrial fibrillation developed after CABG in 156 patients (15.8%). Patients with POAF were generally older (P = 0.001) and presented more often with comorbidities including congestive heart failure (P = 0.001), hypertension (P = 0.001), peripheral vascular disease (P = 0.001), hyperlipidemia (P = 0.009), and renal failure (P = 0.001). Five-year mortality was observed in 23 (2.3%) patients. Patients with POAF had higher five-year mortality rate than those without POAF. Multivariate logistic analysis showed that AF after surgery has a strong effect on mortality (HR, 3.3; 95% CI, 0.04-10.8, P = 0.04) and morbidity rates (HR, 4.0; 95% CI, 2.35-6.96, P = 0.001).

**Conclusions:**

Postoperative atrial fibrillation strongly predicts higher long-term mortality and morbidity following coronary artery bypass graft.

## 1. Background

New onset atrial fibrillation (AF) is the most common arrhythmia following coronary artery bypass grafting (CABG) surgery (1). Reported incidence of AF after CABG surgery varies from 25% to 40% occurring usually between second and fourth postoperative days (2-6). Although this arrhythmia is self-limiting in most cases, it can cause hemodynamic disturbances, increased medical costs, and prolonged hospitalization (7-10). Short-term effects of postoperative AF (POAF) are well known but its long-term consequences are not well established or clearly understood. Most of the data related to the effects of POAF on long-term mortality and morbidity rates are limited to first five years after surgery; few studies have reported very late effects of post-CABG AF (11-13).

## 2. Objectives

The objective of this study was to evaluate the impact of POAF on very late (> 5 years) mortality and morbidity rates after isolated CABG. 

## 3. Patients and Methods

### 3.1. Study Protocol and Patient Population

In this retrospective cohort study, we reviewed our database of Adult Cardiac Surgery to identify patients who underwent isolated CABG between October 2004 and October 2006 and had more than five years follow-up. Patients with history of preoperative AF, permanent pacemaker or implantable cardioverter-defibrillator implantations, and thyroid diseases were excluded from the study.

Our database contained detailed information on patient demographics, preoperative risk factors, operation details, postoperative hospital course, and morbidity and mortality outcomes. These data consisted of gender, age, height, and weight of patients as well as history of hypertension, diabetes, dyslipidemia, smoking, hypothyroidism, and asthma. We also evaluated the patients for other arrhythmic disorders, percutaneous coronary intervention, cardiovascular disease, angiography data, left ventricular dysfunction, ventricular hypertrophy, and reports of bypass surgery. Survival was measured as time (in day) to either death or last follow up. A written informed consent was obtained from all participants and the local Ethics Committee approved the study protocol.

Post-CABG AF was defined according to established criteria of STS (12). It was our general practice to restore sinus rhythm in most patients within 24-48 hours after the onset of POAF using antiarrhythmic drugs (AADs) or by employing electrical cardioversion. If medical therapy resulted in reestablishment of sinus rhythm or control of ventricular rate and the patient was asymptomatic, the medication continued for 6 weeks. In persistent AF with unsuccessful rhythm cardioversion, warfarin was also administrated and patients were discharged on warfarin (in the absence of any contraindication) and referred to cardioversion 4 to 6 weeks later. In the absence of AF recurrence, antiarrhythmic drugs were discontinued. Causes of death were recognized by a review of hospital records, death certificates, and autopsy reports. All patients were visited in six months intervals. The incomplete data were followed up by telephone contact. At each visit, patients were monitored for symptom changes, myocardial infarction, heart failure, embolic cerebrovascular accident, pulmonary emboli, cardiac interventions, medications, and cardiac rhythm (AF episodes). 

### 3.2. Statistical Analysis

The data were recorded in SPSS 17 for windows (SPSS Inc. Chicago, IL, USA). Continuous variables are presented as mean ± SD. The Student’s t-test was employed to compare data between the two groups with a normal distribution. Otherwise, a non-parametric Mann-Whitney U test was employed. To statistically evaluate isolated effects of POAF on mortality rate, a multivariate Cox proportional hazard model was constructed for related survival time as a function of POAF, adjusted for 13 covariates. Adjusted hazard ratios (HRs) along with 95% confidence intervals (CI) were computed for POAF and 13 covariates. Additionally, Kaplan-Meier product-limit estimates were generated to provide in time survival estimates at post-operative points. Provided estimates for POAF status were compared for equality using log-rank tests. A P value < 0.05 was considered statistically significant.

## 4. Results

### 4.1. Patient Characteristics

Study population consisted of 989 post-CABG patients with more than 5-year follow-up. The mean age at the time of cardiac surgery was 60 ± 10 years from which 87% were men. Hypertension was manifested in 50%, dyslipidemia in 45%, and diabetes in 31% of patients, and 32% were smokers. Mean ejection fraction was 44 ± 8.0% in the study population. Overall incidence of AF in whole study population was 15.8% (n = 156).

### 4.2. Baseline Characteristics of the Patients With and Without Postoperative Atrial Fibrillation

Demographic and clinical characteristics of the study population by AF status were summarized in Table 1. Patients with POAF were older than patients without POAF (65 ± 7.0 vs. 59 ± 11.0, P = 0.001) and presented with more severe symptoms (expressed as NYHA functional class) (2.4 ± 0.9 vs. 2.1 ± 0.7, P = 0.001). The prevalence of comorbidities including hypertension (67% vs. 47%, P = 0.001), hyperlipidemia (50% vs. 39%, P = 0.009), renal failure (15% vs. 2.0%, P = 0.001), peripheral vascular disease (19% vs. 4.0, P = 0.001), and chronic obstructive lung disease (8.0% vs. 4.0%, P = 0.05) were also significantly higher in patients with POAF. However, the prevalence of smoking and diabetes were similar in both groups. Left ventricular function was significantly lower in patients with POAF (43.0 ± 8.0% vs. 46.0 ± 7.0%, P = 0.001). Left atrial dimension was also significantly greater in POAF group (37 ± 9.0 vs. 35 ± 5.0 mm respectively, P = 0.001). Patients without POAF were medicated more than POAF patients by beta-blockers (80% vs. 64%, P = 0.001), angiotensin converting enzyme inhibitors (69% vs. 58%, P = 0.004), and statins (61% vs. 48%, P = 0.002).

**Table 1.** Demographic and clinical characteristics by postoperative atrial fibrillation

	POAF ^a^ (n = 156)	No POAF (n = 833)	*P* value
Age, year	65 ± 7.0	59 ± 11.0	0.001
Male	129 (83 %)	731 (87 %)	0.085
LVEF^a^	43.0 ± 8.0	46.0 ± 7.0	0.001
LA^a^ dimension, mm	37 ± 9.0	35 ± 5.0	0.001
Hypertension	105 (67 %)	391 (47 %)	0.001
Hyperlipidemia	78 (50 %)	323 (39 %)	0.009
Smoking	51 (33 %)	289 (35 %)	0.630
Diabetes	54 (35 %)	255 (31 %)	0.300
Renal failure	24 (15 %)	17 (2.0 %)	0.001
Peripheral vascular disease	30 (19 %)	34 (4.0 %)	0.001
Myocardial infarction	57 (36 %)	357 (43 %)	0.140
Obstructive lung disease	12 (8.0 %)	34 (4.0 %)	0.050
Percutaneous revascularization	12 (8.0 %)	136 (16.0 %)	0.006
Preoperative drugs			
Betablockers	99 (64 %)	663 (80 %)	0.001
ACE ^a^ inhibitors	90 (58 %)	578 (69 %)	0.004
Calcium blockers	45 (29 %)	272 (33 %)	0.350
Statins	75 (48 %)	510 (61 %)	0.002

^a^ Abbreviations: ACE: Angiotensin-Converting Enzyme ; LVEF=Left Ventricular Ejection Fraction; LA: Left Atrium; POAF: Postoperative Atrial Fibrillation

Considering intraoperative factors, intra-aortic balloon pump (IABP) was more inserted in POAF patients compared to patients without POAF (1.9% vs. 0%, P = 0.004). Patients with POAF experienced more stroke during surgery than those in other group (5.8% vs. 0%, P = 0.001). Evaluation of discharge medications showed that patients with POAF were more likely to be discharged on beta-blocker, calcium channel blocker, warfarin, and amiodarone (Table 2). 

**Table 2.** Discharge medications according to postoperative atrial fibrillation

	POAF ^a^ (n = 156 )	No POAF (n = 833 )	*P* value
Beta-blockers	156 (100 %)	782 (94 %)	0.001
Calcium Chanel- blocker	30 (19 %)	85 (10 %)	0.001
ACE ^a^ inhibitors	132 (85 %)	697 (84 %)	0.769
Statin	108 (71 %)	680 (82 %)	0.002
Warfarin	36 (23 %)	34 (4.0 %)	0.001
Aspirin	150 (96 %)	799 (96 %)	0.89
Amiodarone	51 (33 %)	34 (4.0 %)	0.001

^a^ Abbreviations: ACE: Angiotensin-Converting Enzyme; POAF: Postoperative Atrial Fibrillation

### 4.3. Association between New Onset POAF and Long-Term Mortality and Morbidity

Long-term mortality rate was 2.3% in whole population (5.8% in group with POAF vs. 1.7% in group without POAF). New onset POAF was associated with higher long-term mortality (HR = 3.6; 95% CI: 1.52-8.43; P = 0.005). In patients with POAF, myocardial infarction (55%, n = 5), cerebrovascular accident (33%, n = 3), and congestive heart failure (12%, n = 1) were diagnosed as etiologies of the death. 

We determined morbidity as incidence of one complication during the follow up; thereby the incidence of long-term morbidity was 12.5% (25% in group with POAF vs. 10% in group without POAF). Postoperative late outcome were summarized in Table 3. The frequency of postoperative complications, such as stroke (HR = 7.5, 95% CI: 3.84-14.52, P = 0.001) and myocardial infarction (HR = 6.66, 95% CI: 5.74-7.73, P = 0.001) were higher in patients with POAF compared to those without POAF. Patients with POAF remained hospitalized longer than patients without POAF (15 days vs. 11 days, P = 0.001). Long-term morbidity was also higher in POAF patients than that of in the second group (HR = 2.9; 95%CI: 1.91-4.49, P = 0.001). In multivariable analysis, atrial fibrillation remained as an independent predictor of very late mortality. Other predictors included history of HTN and myocardial infarction (Table 4). Multivariate analysis in presence of other confounding factors or other covariate affecting morbidity also showed that POAF is an independent predictor of long-term morbidity. Other predictors for morbidity were NYHA classification, smoking status, renal failure, and history of myocardial infarction (Table 5). 

**Table 3.** Postoperative complications by presence of atrial Fibrillation

	POAF ^a^ (n = 156)	No POAF (n = 833)	*P* value
Heart failure	9 (6.0 %)	34 (4.0 %)	0.343
Postoperative stroke	21 (13.0 %)	17 (2.0 %)	0.001
Myocardial infarction	9 (6.0 %)	0 (0.0 %)	0.001
Renal failure	3 (2.0 %)	34 (4.0 %)	0.192
Length of hospital stay, day	15 ± 2.5	11 ± 4.0	0.001

^a^ Abbreviation: POAF: Postoperative Atrial Fibrillation

**Table 4.** Predictors of mortality after coronary artery bypass surgery in multivariate analysis

Variable	Adjusted Hazard Ratio (95% CI^a^)	*P* value
Atrial fibrillation	3.3 (0.04-10.8)	0.040
Female gender	1.2 (0.11-13.0)	0.870
Age	0.94 (0.88-1.00)	0.050
classification	0.98 (0.57-1.67)	0.950
Smoking status	1.74 (0.52-5.86)	0.360
Diabetic mellitus	0.49 (0.13-1.72)	0.260
Renal failure	0.78 (0.08-7.52)	0.830
Peripheral vascular disease	1.18 (0.16-8.27)	0.860
Myocardial infarction	35 (4.35-277.0)	0.001
Percutaneous revascularization	2.5 (0.47-12.70)	0.280
Hypertension	3.7 (1.25-11.40)	0.010
Hyperlipidemia	1.58 (0.54-4.65)	0.400

^a^ Confidence Interval

**Table 5.** Predictor of morbidity after coronary artery bypass surgery in multivariate analysis

Variable	Adjusted Hazard Ratio(95% CI ^a^)	P value
AF	4.0 (2.35-6.96)	0.001
Female gender	1.0 (1.41-2.80)	0.880
Age	1.0 (0.98-1.03)	0.370
NYHA ^b^	1.4 (1.07-1.81)	0.010
Hypertension	1.0 (0.64-1.53)	0.990
Hyperlipidemia	6.8 (0.66-12.65)	0.001
Smoking status	3.5 (2.19-5.69)	0.001
Diabetic mellitus	1.5 (0.89-2.67)	0.110
Renal failure	4.8 (1.27-18.01)	0.020
Peripheral vascular disease	0.6 (0.20-1.70)	0.330
Myocardial infarction	1.9 (1.19-2.95)	0.006
Percutaneous revascularization	0.5 (0.18-1.13)	0.090

^a^ Confidence Interval

^b^ **Abbreviation: NYHA: New York Heart Association**

## 5. Discussion

### 5.1. Major Findings

The present study clearly demonstrated that new onset POAF increases very late mortality after isolated CABG and is associated with relevant thromboembolic sequel. The incidence of POAF (15.8%) was consistent with which reported earlier in literature. 

### 5.2. Postoperative Atrial Fibrillation and Long-Term Mortality and Morbidity

Few studies have evaluated the effect of POAF on long-term survival after CABG. In a study of 6475 patients, Villareal et al. (4) reported a higher risk of late mortality in patients with POAF after isolated CABG (OR, 3.4; 95%CI 1.6-7.5). However, age was entered as a dichotomous variable in their multivariate model which could have biased the effect of age on mortality rate. Mariscalco et al.(11) similarly reported an increased risk of long-term mortality in patients with POAF (HR = 2.99; 95% CI: 2.33-3.84). Results of this study were also confounded by 8% patient loss during follow-up and inclusion of patients with preoperative paroxysmal or persistent AF. Filardo et al.(12)reported similar findings from 6899 isolated CABG patients. A significantly lower ten-year survival was observed in patients who developed POAF compared to those without POAF (HR = 1.29; 95% CI: 1.16-1.45). We similarly confirmed negative survival effect of POAF in CABG patients. In the present study, POAF increased the risk of death by 3.6 in CABG patients.

The frequency rates of postoperative complications such as ischemic stroke, myocardial infarction, and prolonged hospital stay were increased in patients with POAF. In the present study, the risk of ischemic stroke were increased by 7.5 times in patients with POAF . The increased risk of cerebrovascular accident occurred despite the fact that AF patients were adequately managed by oral anticoagulants. Higher risk of the stroke in AF patients may be explained by impaired hemodynamics associated with reduced ventricular filling and circulatory stasis in the left atrium that rendered patients susceptible to stroke and other embolic events. This complication also adversely affected patient’s survival because stroke was responsible for at least one-third of late mortalities. In addition, the increased morbidity related to the stroke always associated with increased length of hospital stay. Although we did not measure the exact role of ischemic stroke in prolongation of hospital stay, this complication may have importantly contributed to longer hospital stay observed in AF group (15 days vs. 11 days, P < 0.001). 

Another important finding of this study was that POAF is an age-independent risk factor for postoperative MI with an adjusted hazard ratio of 6.6 (95% CI: 5.74-7.73). This complication had marked effect on poor outcome and mortality of patients as late deaths secondary to MI were frequent in this study (55%). A similarly increased risk of MI in POAF patients has been demonstrated in two previous studies. In the study of Ahlsson and associates, a higher rate of death due to MI was reported (6.7% vs. 3.0%, P = 0.041) (13). Mariscalco and colleagues similarly reported an increased risk of death due to embolic events (MI and stroke) in CABG patients with POAF (HR, 4.33; 95%CI 1.78–10.52, P = 0.001) (11). Currently, there is no clear explanation for increased cardiovascular mortality due to embolic events in patients with POAF. Is it related to occurrence of late AF? Currently, there are some data showing that patients experienced POAF are more prone to develop AF during follow-up (13). An episode of POAF was the strongest independent risk factor for development of late AF with an adjusted risk ratio of 8.31 (95%CI, 4.20-16.43). Another hypothesis for higher rate of late mortality in POAF patients might be related to the fact that, POAF is associated with an increased incidence of several comorbidities including advanced age, heart failure, renal failure, chronic lung disease, and peripheral vascular disease. 

### 5.3. Study Limitations

Prevalence of postoperative AF was 15.8%, which is significantly lower than other reports. This low prevalence may be explained by exclusion of patients with valvular disease and those with history of the preoperative AF. In this cohort study of patients who underwent CABG, the prevalence of POAF after CABG was 15.8% in our population and was associated with increased mortality and morbidity rates even more than five years after surgery. Further researches may focus on development of more effective methods for prevention as well as identification of high risk patients who are susceptible to new onset POAF and thereby better prophylactic plans could be designed for such patients. 
